# Microstructure and Mechanical Properties of Al–(12-20)Si Bi-Material Fabricated by Selective Laser Melting

**DOI:** 10.3390/ma12132126

**Published:** 2019-07-02

**Authors:** Shikai Zhang, Pan Ma, Yandong Jia, Zhishui Yu, Rathinavelu Sokkalingam, Xuerong Shi, Pengcheng Ji, Juergen Eckert, Konda Gokuldoss Prashanth

**Affiliations:** 1 School of Materials Engineering, Shanghai University of Engineering Science, Shanghai 201620, China; 2 Laboratory for Microstructures, Institute of Materials, Shanghai University, Shanghai 200444, China; 3 Advanced Materials Processing Laboratory, Department of Metallurgical and Materials Engineering, National Institute of Technology, Tiruchirappalli, Tamil Nadu 620015, India; 4 Erich Schmid Institute of Materials Science, Austrian Academy of Sciences, Jahnstraße 12, A-8700 Leoben, Austria; 5 Department of Materials Science, Montanuniversität Leoben, Jahnstraße 12, A-8700 Leoben, Austria; 6 Department of Mechanical and Industrial Engineering, Tallinn University of Technology, Ehitajate tee 5, 19086 Tallinn, Estonia

**Keywords:** Al–Si, selective laser melting (SLM), microstructure, mechanical properties

## Abstract

In this study, a combination of Al–12Si and Al–20Si (Al–(12-20)Si) alloys was fabricated by selective laser melting (SLM) as a result of increased component requirements such as geometrical complexity and high dimensional accuracy. The microstructure and mechanical properties of the SLM Al–(12-20)Si in as-produced as well as in heat-treated conditions were investigated. The Al–(12-20)Si interface was in the as-built condition and it gradually became blurry until it disappeared after heat treatment at 673 K for 6 h. This Al–(12-20)Si bi-material displayed excellent mechanical properties. The hardness of the Al–20Si alloy side was significantly higher than that of the Al–12Si alloy side and the disparity between both sides gradually decreased and tended to be consistent after heat treatment at 673 K for 6 h. The tensile strength and elongation of the Al–(12-20Si) bi-material lies in between the Al–12Si and Al–20Si alloys and fracture occurs in the Al–20Si side. The present results provide new insights into the fabrication of bi-materials using SLM.

## 1. Introduction

The characteristic properties of aluminum, such as high strength and stiffness-to-weight ratio, good formability, good corrosion resistance, and recycling potential make it an ideal candidate for automobile and aerospace applications [1,2]. The use of aluminum reduces the dead weight of the components, leading to more fuel-efficient vehicles, lower energy consumption and less air pollution [1,2,3,4]. Among the families of Al-based alloys, Al–Si is one of the most commonly used cast alloys because of its advantages concerning fluidity [5,6]. With increasing silicon content, especially from hypoeutectic to hypereutectic composition, the strength, tribological properties and corrosion resistance of the alloy increases significantly. However, the ductility, thermal conductivity and machinability show the opposite trend [7,8,9,10]. Moreover, the performance requirements of different parts of any particular component are diverse and can be demanding based on the stringent requirements for reduced waste and/or minimal use of resources. Such stringent usage and heavy demands often lead to the use of new alloys replacing traditional alloys produced by casting [11,12]. Hence, parts with different Si contents may be preferred depending on the application, where the outer surface of the components may require good tribological properties (higher Si content) and the core of the part should have good ductility (lower Si content). Hence, application of new methods to produce such components is of primary interest.

In comparison with traditional processes, additive manufacturing (AM) technologies offer significant benefits, such as near-net-shape production capabilities, superior design and geometrical flexibility, reduced tooling, shorter cycle time for design and manufacturing, as well as material, energy and cost efficiency. In particular, selective laser melting (SLM) is an AM process in which functional, complex parts are produced by selectively melting consecutive layers of powder with a laser beam [13,14,15,16]. In SLM, layers of atomized powder are spread sequentially on a building platform. The powder bed is then melted selectively by a laser beam. The melt pool is cooled rapidly by the underlying substrate or the previously built metal layer. The parameter optimization is rather simple based on an empirical rule of thumb considering the energy density, which strongly depends on laser power, laser scan speed, layer thickness, hatch distance and laser spot size [16,17]. In general, the parameters of the process are decided by the defect content in the material rather than using the melting point or solidification range as reference [18]. The flexibility in parameter selection offered by SLM enables the exploration of a wide spectrum of possibilities for creating novel alloys or even metal–metal composites with unique microstructures [18,19,20,21,22]. Heat treatments at high temperatures can promote the coalescence of second phases and change their distribution [23,24]. In this research, Al–(12-20)Si bi-materials were fabricated by SLM and heat treated at different temperatures. The microstructure and the mechanical properties of the Al–(12-20)Si bi-material fabricated by SLM were systematically studied.

## 2. Experimental Methods

**Figure 1:**
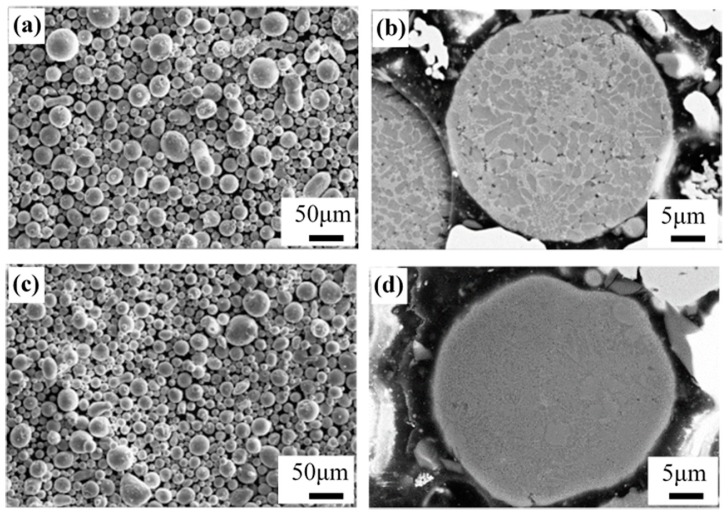
Microstructures of (**a**,**b**) Al–12Si, and (**c**,**d**) Al–20Si gas atomized powders.

Spherical gas atomized Al–12Si and Al–20Si powders with an average particle size of 40 μm were used for the experiments, as illustrated in Figure 1. SLM solutions SLM280^HL^ device equipped with a Nd-YAG laser was used for preparing the Al–12Si and Al–20Si bi-materials. Both Al–12Si and Al–20Si are manufactured using the same SLM processing conditions. The processing parameters have been optimized as follows: the laser power was 320 W, laser scan speed was 1455 mm/s, the powder layer thickness was 50 μm, the hatch spacing was 110 μm and the hatch style rotation was 73°. High purity argon gas was used to ventilate the chamber before and during the building process to maintain a low concentration of oxygen. For comparison, the Al–12Si and Al–20Si alloys were also fabricated separately by SLM using the same experimental parameters. The SLM samples were subsequently heat-treated at 473 ± 1 K, 573 ± 1 K and 673 ± 1 K for 6 h and cooled to room temperature by furnace cooling.

**Figure 2:**
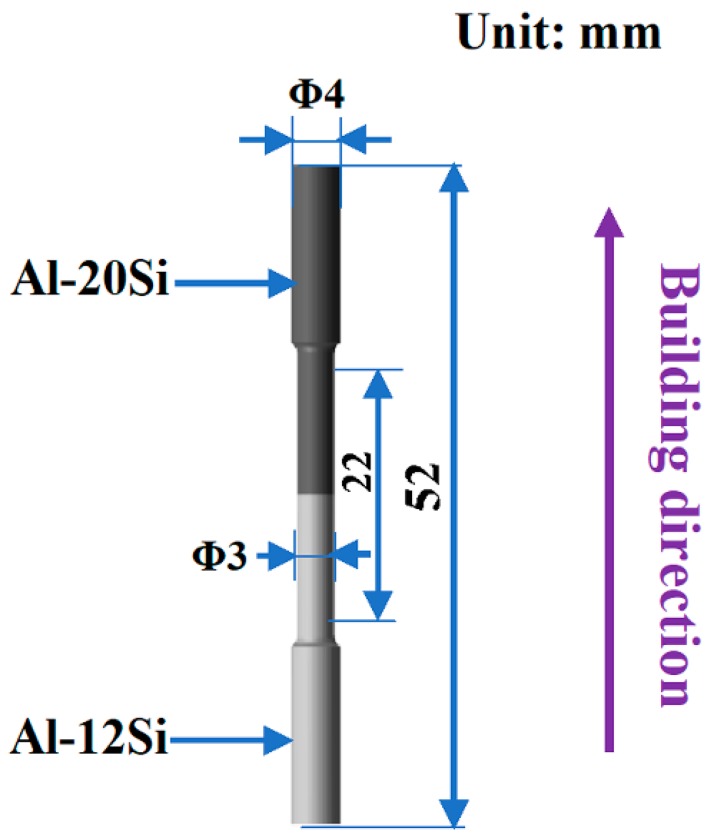
Schematic diagram of tensile samples of Al–(12-20)Si bi-material.

The microstructures and fracture morphologies of the SLM and heat-treated samples were characterized by optical microscopy (OM) using an Olympus optical microscope (Olympus, Hamburg, Germany) and by scanning electron microscopy (SEM) using a Gemini 1530 microscope (Jeol, München, Germany) operated at 20 KV after etching with 0.5% HF solution (0.5% HF, 99.5% H_2_O, in vol %). Vickers microhardness measurements were carried out using a HV-1000 Z-type digital microhardness tester (Stuers, Willich, Germany) with 100 g load and 15 s dwell time. Tensile tests were performed with an INSTRON 5569 testing machine (INSTRON, Darmstadt, Germany) along the building direction at a strain rate of 1 × 10^−4^ s^−1^, as shown in Figure 2. In order to ensure reproducibility of the tensile results, the mechanical properties were determined by mathematically averaging the measurement results of at least six samples. 

## 3. Results and Discussion

**Figure 3:**
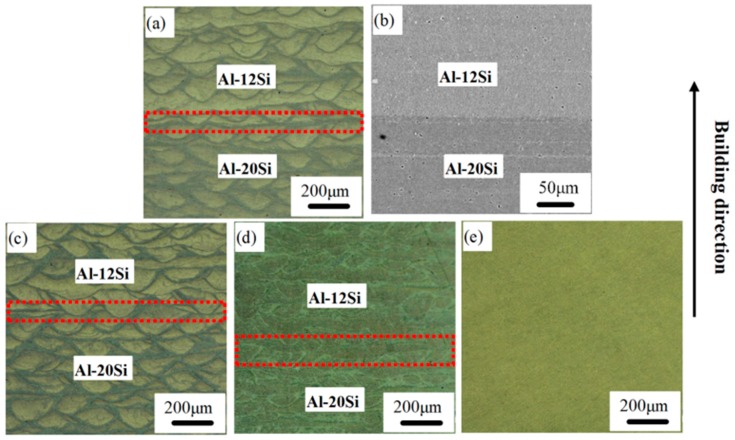
Microstructure of Al–Si bi-materials fabricated by selective laser melting (SLM) and heat treatment: (**a**,**b**) SLM; (**c**) SLM + 473 K/6 h; (**d**) SLM + 573 K/6 h; (**e**) SLM + 673 K/6 h.

Figure 3 shows OM and SEM images of the Al–12Si and Al–20Si bi-materials fabricated by SLM with and without heat treatment at different temperatures. The morphology of the as-SLM material is shown in Figure 3a,b. The boundary between Al–12Si and Al–20Si is distinct due to differences in Si content (marked by dotted lines). As can be seen in Figure 3a, the average width of the melt pool is about 160 μm, and the penetration depth is around 90 μm along the Al–12Si side. On the other hand, the average width of the melt pool is approximately 204 μm, and the penetration depth is about 87 μm along the Al–20Si side. This suggests that Al–20Si exhibits elongated melt pools with a typical length-to-width aspect ratio of about 2.35:1. Concerning the geometrical characteristics, a single track of the melt pool dimensions, including the width and depth, is correlated with the energy input. As more Si exists in the Al–20Si alloy than in the Al–12Si alloy and the experimental parameters used are the same for both, the additional melting of 8 wt % more Si in the Al–20Si alloy requires more energy. Upon consumption of additional energy for melting, the depth of the melt pool in Al–20Si is reduced; however, the width is increased compared to the Al–12Si side and the length of the melt pool remains constant, suggesting shallow melt pool characteristics on the Al–20Si side [25,26].

Because of Marangoni convection [26], a large amount of Si segregates at the hatch overlaps and/or boundaries are present in the material and this phenomenon is more distinct at the Al–20Si side. The primary silicon phase is driven by the fluid flow in the melt pool and starts to solidify along the low temperature region of the melt pool. Thus, the primary silicon phase is concentrated at the contour regions of the melt pool and along the hatch overlaps. The interfaces are still visible and there are no significant variations in the microstructure after heat treatment at 473 K for 6 h (Figure 3c). Compared with Figure 3a–c, the interface in Figure 3d is rather blurred; moreover, the contour regions also become unclear and it is difficult to distinguish the track cores and the hatch overlaps. Finally, both the heat affected zone and the interface disappear after heat treatment at 673 K for 6 h, as shown in Figure 3e. Therefore, with increasing heat treatment temperature, the interface becomes indistinct and the microstructure becomes uniform. When heat treated at 673 K, the diffusion of Si atoms as well as growth of Si particles are accelerated, both promoting the uniform distribution of Si in the Al matrix [23,25].

**Figure 4:**
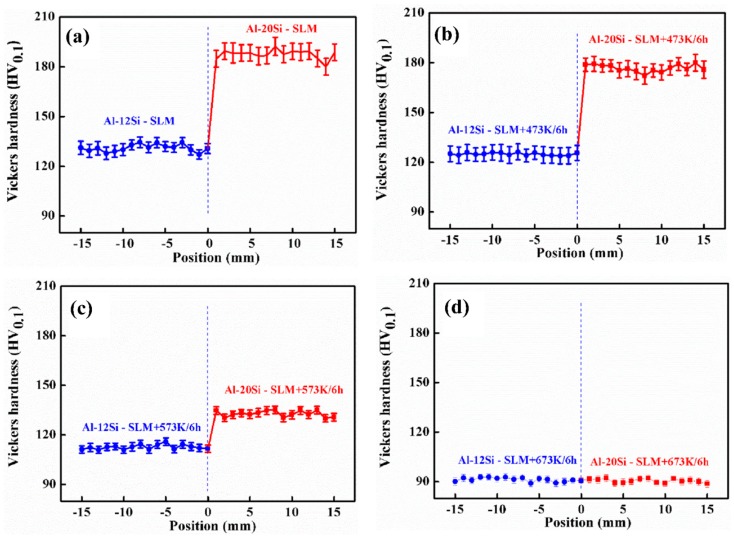
Vickers hardness of Al–(12-20)Si bi-materials: (**a**) SLM; (**b**) SLM + 473 K/6 h; (**c**) SLM + 573 K/6 h; (**d**) SLM + 673 K/6 h.

Figure 4 displays the hardness profile of the Al–Si bi-material as a function of the distance from the interface after SLM and after heat treatment at different temperatures. As observed in Figure 4a, the average Vickers hardness of the Al–20Si side is 188 HV_0.1_ and 131 HV_0.1_ for the Al–12Si side. The SLM-processed samples demonstrate superior hardness compared to alloys with the same composition fabricated with other methods [27,28]. As expected, the hardness of the Al–20Si alloy is significantly higher than at the Al–12Si side because of its higher Si content [27,28].

It is well-known that both the hardness and the strength of Al-based alloys processed by SLM decrease with heat treatment [24,27]. Similarly, in the Al–(12-20)Si bi-material, the hardness decreases after thermal treatment (Figure 4b–d). The hardness of Al–12Si decreases from 131 HV_0.1_ (as-prepared SLM) to 125 HV_0.1_, 113 HV_0.1_ and 91 HV_0.1_ after heat treatment at 473 K, 573 K and 673 K for 6 h, respectively. Meanwhile, along the Al–20Si side, the hardness decreases from 188 HV_0.1_ (as-prepared SLM) to 177 HV_0.1_, 133 HV_0.1_ and 91 HV_0.1_ after heat treatment at 473 K, 573 K and 673 K for 6 h, respectively. Heat treatment at 673 K causes the hardness on both the Al–12Si and the Al–20Si side to become similar, suggesting that there is enough time for the Si atoms to diffuse from one side to the other and to reach an equilibrium state [27,29]. Further heat treatment will further reduce the hardness, but it will remain similar on both sides. Hence, the hardness profile for the 673 K heat treated sample remains flat without any variations from the Al–12Si to the Al–20Si side.

**Figure 5:**
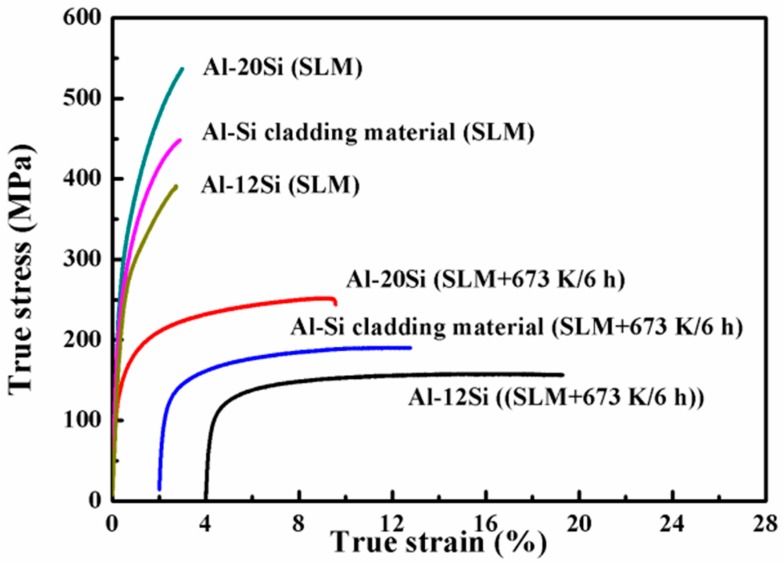
True stress–strain curves of Al–Si alloys and Al–(12-20)Si bi-materials.

Representative room temperature stress–strain curves of the quasistatic tensile tests of the SLM-fabricated Al–Si alloys and the bi-material are presented in Figure 5. The as-prepared Al–12Si alloy exhibits an ultimate tensile strength (UTS) and yield strength (YS) of approximately 380 MPa and 260 MPa, respectively, with a room temperature plasticity of about 2.5%. Most of the SLM samples have excellent tensile strength, significantly better than their cast counterparts [27]. For instance, as-cast Al–12Si normally has a tensile strength of approximately 200 MPa [27,28,29,30,31,32,33,34] and in the present as-prepared condition, the SLM samples exhibit a strength of approximately 380 MPa. Some studies introduced ultrasonic melt treatments and equal-channel angular extrusions in order to refine the grains and enhance their mechanical properties [31,32]. After such treatment, the UTS was about 309 MPa and 230 MPa, respectively. Moreover, conventional powder metallurgy techniques were used to fabricate Al–12Si alloys, and the tensile strength was observed to be 174 MPa and 220 MPa, respectively, after hot pressing and hot extrusion [33]. Similarly, the strength of Al–20Si SLM parts is higher than the values obtained by most manufacturing methods [34,35,36,37]. Yoon et al. [36] reported an ultimate tensile strength of the Al–20Si alloy after extrusion and equal channel angular pressing of about 350 MPa. Furthermore, high pressure solidified Al–20Si samples show an UTS of 365 MPa after high pressure solidification [37].

Figure 5 reveals that the YS and UTS of the bi-materials are about 450 MPa and 300 MPa, respectively, with approximately 2.2% ductility. Moreover, the Al–(12-20)Si bi-material exhibits mechanical properties that are between those of the Al–12Si and Al–20Si alloys. Since the melt pool is very small (a few micrometers in length), depending on the powder characteristics and on the processing parameters used, the cooling rates during SLM can reach very high values (1 × 10^3^–1 × 10^6^ K/s), which can increase the nucleation rate and suppress grain growth [13,38]. Correspondingly, this high strength can be attributed to the very fine microstructure as well as the formation of a supersaturated solid solution during the process.

**Figure 6:**
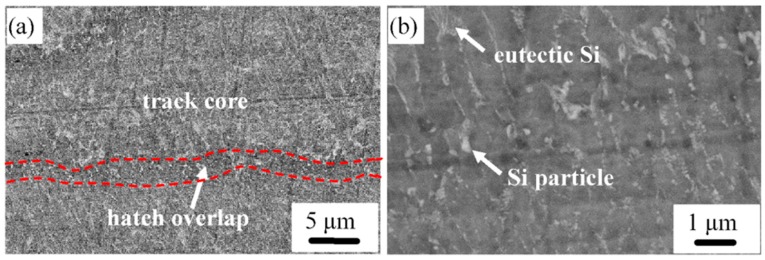
Longitudinal microstructure of the Al–12Si side in the Al–(12-20)Si bi-material (**a**) low magnification and (**b**) high magnification.

The longitudinal microstructure of the SLM bi-materials is shown in Figure 6, revealing that the morphology of the Al–12Si side in the Al–(12-20)Si bi-material is parallel to the building direction. The microstructure is anisotropic and is composed of track cores and hatch overlaps. Fibrous eutectic Si is located around α-Al and presents a columnar morphology in the track cores. In contrast, the α-Al phase is surrounded in circles with Si particles at the relatively narrow hatch overlaps.

**Figure 7:**
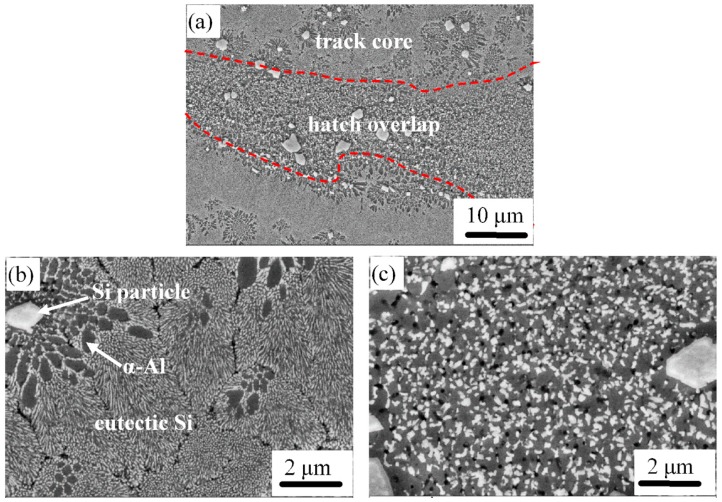
Longitudinal microstructure of the Al–20Si side in the Al–(12-20)Si bi-material, where (**a**) shows the microstructure along with both track cores and hatch overlaps; (**b**) microstructure along with the core of the tracks; and (**c**) microstructure along with the hatch overlaps.

The microstructure in Figure 7 along the Al–20Si side is inhomogeneous and shows typical SLM features like track core morphology with hatch overlaps. The track cores consist of fibrous eutectic (Al + Si), Si particles and a small quantity of α-Al phase, as illustrated in Figure 7b. The size of the fibrous eutectic Si is approximately 500 nm. The grain boundaries can be seen clearly in Figure 7. The hatch overlap region (Figure 7c) is composed of Si particles with an average size of 300 nm. Grain refinement leads to reduction of the distance between the Si particles, which can give a considerable contribution to the strength because the increased number of Al–Si interfaces can effectively reduce the movement of dislocations [39,40]. As discussed above, such microstructural modification obtained through SLM processing can enhance the mechanical properties. Similar observations were made for SLM-processed Al–Si based alloys [27,28].

The bi-material heat treated at 673 K for 6 h was further investigated in order to gain insight about the effect of heat treatment on the tensile properties. The UTS decreases to approximately 200 MPa and the YS decreases to approximately 125 MPa. However, the elongation increases to 9.0% for the heat treated bi-material. Table 1 lists the tensile stress–strain data of the Al–12Si alloy, the Al–20Si alloy and the Al–(12-20)Si bi-material after SLM fabrication and after heat treatment at 673 K for 6 h. The comparison reveals that the Al–(12-20)Si bi-material after heat treatment has mechanical properties that are still between the tensile properties of the Al–12Si alloy and the Al–20Si alloy under heat-treated conditions.

**Figure 8:**
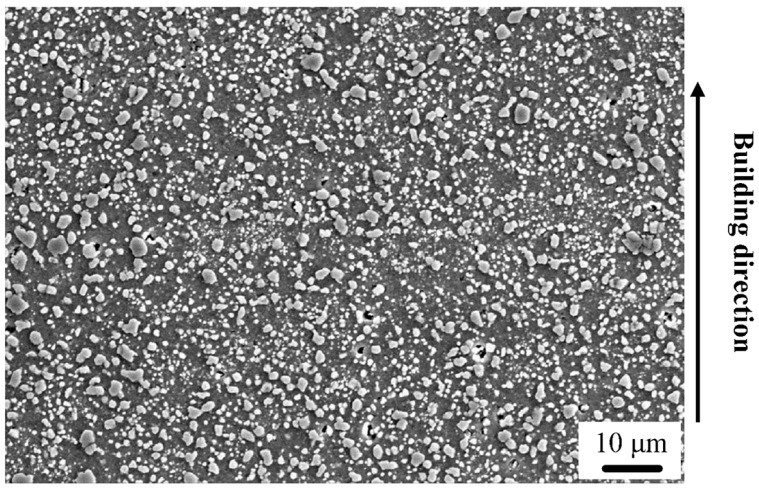
Microstructure of the Al–(12-20)Si bi-material after heat treatment at 673 K for 6 h.

Figure 8 presents the morphology of the Al–Si bi-material after heat treatment at 673 K for 6 h. Due to different Si contents, Si particles diffuse from the Al–20Si side to the Al–12Si side during heat treatment. Moreover, with increased heat treatment temperature, the melt pool boundaries and the heat affected zones disappear. In addition, the Si particles become coarse and their average size increases to approximately 1.31 μm.

During heat treatment at 673 K, the number of Si particles decreases as a result of diffusion of the Si particles from the Al–20Si to the Al–12Si side as well as due to particle coalescence and growth. The distance between adjacent particles becomes large, and upon loading, the dislocations can move easily for long distances without obstacles. Furthermore, the decrease in the number of Si particles and the increase in size induce a reduction of localized stress or strain. The residual stresses that are built up during the SLM process are also relieved during the thermal treatment. Accordingly, the heat treated bi-material is characterized by decreased strength and large elongation.

**Figure 9:**
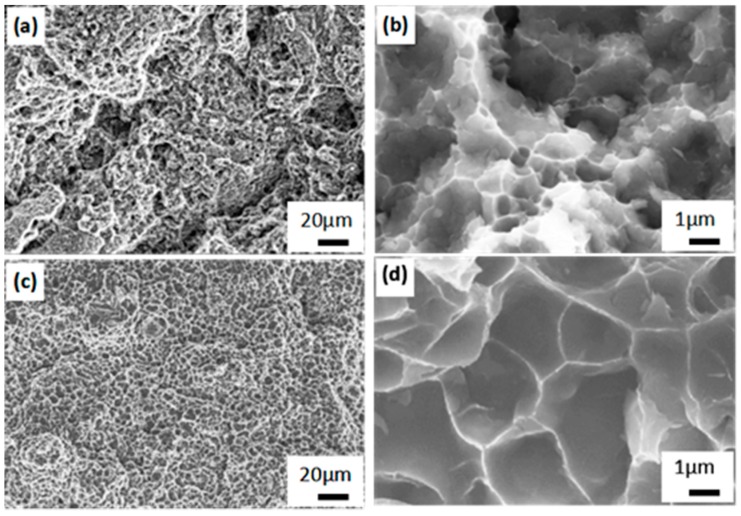
Fracture surface of tensile samples: (**a**,**b**) as-prepared SLM, (**c**,**d**) SLM and heat treated at 673 K for 6 h.

Figure 9 depicts the fracture morphologies of the Al–(12-20)Si bi-material both in as-built as well as heat treated conditions. It can be observed that the failure occurs along the Al–20Si side rather than in the interfacial zone, which demonstrates that good bonding is established between Al–12Si and Al–20Si by the SLM process. Figure 9a,b shows typical fracture surfaces of the Al–(12-20)Si bi-material after SLM processing, revealing a layered structure. Some cleavage planes surrounded by tearing ridges are also visible. Moreover, the dimples are not continuous and are mixed with brittle fracture characteristics, indicating a typical quasi-cleavage fracture mode. The fracture morphologies of the bi-materials after heat treatment are shown in Figure 9c,d. A large number of dimples can be observed, and the size of the dimples is considerably larger compared with Figure 9a,b (in the as-prepared SLM material). The observation of more dimples is well correlated with larger elongation after heat treatment and points to a ductile failure mode.

## 4. Conclusions

Al–(12-20)Si bi-materials were manufactured by selective laser melting and were further heat treated at different temperatures. The microstructure and the mechanical properties were investigated, and the main results can be summarized as follows:

(1) The interfaces are well bonded and can be evidently observed in the bi-material after SLM, whereas they become blurry and finally disappear with increased heat treatment temperature. The microstructure is well refined along both sides (the Al–12Si side and the Al–20Si side). The hardness difference between both sides gradually decreases when the samples are heat treated at 673 K and the hardness values tend to be consistent.
(2) The tensile strength and elongation of the bi-material after SLM are between that of as-prepared SLM Al–12Si and Al–20Si alloys, and failure occurs along the Al–20Si side with quasi-cleavage fracture. The average yield strength, ultimate tensile strength and elongation of the Al–Si bi-material after SLM are 300 MPa, 450 MPa, and 2.2% respectively.
(3) The yield strength and the ultimate tensile strength decrease to 100 MPa and 130 MPa, respectively, and the elongation increases to 14.1% after heat treatment at 673 K for 6 h. Failure is characterized by a ductile fracture mode as a result of increased Si particle size and a decreased number of Si particles.

## Figures and Tables

**Table 1:** Mechanical properties of Al–Si alloys and the Al–Si bi-material. UTS = ultimate tensile strength; YS = yield strength.

Composition	Status	UTS (MPa)	YS (MPa)	Elongation (%)
Al–20Si	SLM	500 ± 13	340 ± 9	1.8 ± 0.1
	SLM + 673 K/6 h	240 ± 7	150 ± 3	5.0 ± 0.2
Al–Si bi-material	SLM	450 ± 11	300 ± 9	2.2 ± 0.1
	SLM + 673 K/6 h	200 ± 7	125 ± 3	9.0 ± 0.4
Al–12Si	SLM	380 ± 10	260 ± 7	2.5 ± 0.1
	SLM + 673 K/6 h	130 ± 3	100 ± 3	14.1 ± 0.5
